# Nocturnal enuresis in obese children: a nation-wide epidemiological study from China

**DOI:** 10.1038/s41598-019-44532-5

**Published:** 2019-06-10

**Authors:** Anyi Zhang, Shenghui Li, Yiwen Zhang, Fan Jiang, Xingming Jin, Jun Ma

**Affiliations:** 1 Department of Developmental and Behavioral Pediatrics, Shanghai Children’s Medical Center, Shanghai Jiao Tong University School of Medicine, Shanghai, China; 2 Shanghai Institute of Pediatric Translational Medicine, Shanghai Children’s Medical Center, Shanghai Jiao Tong University School of Medicine, Shanghai, China; 3 MOE-Shanghai Key Laboratory of Children’s Environmental Health, Shanghai Jiao Tong University School of Medicine, Shanghai, China; 4 Shanghai Jiao Tong University School of Medicine, Shanghai, China

**Keywords:** Diseases, Health care

## Abstract

Childhood obesity increases the risk of obstructive sleep apnea syndrome, type 2 diabetes mellitus, cardiovascular abnormalities, and psychological and behavioral disorders. But it is unclear whether obesity is associated with childhood nocturnal enuresis (NE). This study aimed to assess the relationship between childhood obesity and NE in a nationally representative large sample in China. Subjects were enrolled from Urumqi, Chengdu, Xi’an, Hohhot, Wuhan, Canton, Shanghai, and Harbin cities in China in November and December 2005. The survey included 20,987 children aged 5–12 years and they and their caregivers completed questionnaires. Height and weight were measured by school teachers trained in healthcare. According to the WHO child growth standards, obesity was defined as a body mass index >95^th^ percentile of peers with the same age and gender. NE was defined as bed wetting for more than twice a week for 3 consecutive months. Demographic variables were compared among different groups. The prevalence of obesity, asthma, attention-deficit/hyperactivity disorder (ADHD), depressive moods, and snoring were different between the NE and without-NE groups (P < 0.05). The raw odds ratio (OR) for NE and obesity was 1.36 (95%CI = 1.07–1.74; P = 0.013) and the adjusted OR was 1.42 (95%CI = 1.11–1.82; P = 0.005) in the multivariable analysis. When adjusting for co-occurring conditions, the results showed that asthma did not affect the risk of NE (OR = 1.42, 95%CI = 1.11–1.82; P = 0.005), but ADHD (OR = 1.41; 95%CI = 1.10–1.81; P = 0.006) and depressive moods (OR = 1.34; 95%CI = 1.07–1.76; P = 0.012) slightly weakens the association between NE in children and obesity, while snoring weakens the association between obesity and NE and the risk became non-significant (OR = 1.21; 95%CI = 0.94–1.56; P = 0.138). In conclusion, obese children were at a higher risk of incurring NE compared to non-obese children. This association was weaker in children who either snored, had ADHD, or had depressive mood.

## Introduction

Childhood obesity is associated with consuming calories in excess to the body’s energy expenditure, leading to imbalance between energy input and output. Childhood obesity is one of the most prevalent metabolic disorders, with an estimated prevalence of 18% in children at age of 6–11 worldwide^1^. Children with obesity are at increased risk of suffering from obstructive sleep apnea syndrome (OSAS), type 2 diabetes mellitus, cardiovascular abnormalities, and psychological and behavioral disorders (attention deficit/hyperactive disorder, ADHD)^2–4^.

There is also emerging evidence that obesity is associated with childhood nocturnal enuresis (NE)^5,6^. NE is a voiding dysfunction and refers to involuntarily voiding in bed in children beyond the age of 5 years^7^. The effect of obesity on NE has not been extensively studied and the available studies reveal different views on the relationship between obesity and NE. A case-control study showed that obesity and NE were not associated, but obesity and NE were both associated with obstructive sleep apnea^8^. In another community-based study, obese children were more likely to have an overactive bladder (OAB), characterized by wild daytime urgency and incontinence symptoms, but not NE^9^. While other studies have shown the opposite^5,6^, Erdem *et al*. found that 62–86% of the children with voiding dysfunction (including NE) were also diagnosed with obesity^5^. In another cross-sectional study with 281 children and adolescents aged 7–18 from hospital-based samples, children with obesity were at high risk for NE^6^. However, these studies had several limitations. First, most have a small sample size. Second, case-control studies and those with children enrolled from clinics have high selectivity and may draw conclusions that are not representative of the general population of obese children. Third, they did not take confounding factors (like OSAS, ADHD, and depressive moods) into consideration.

Accordingly, we conducted this survey to investigate the underlying association between obesity and NE in a large, representative sample of Chinese children of 5–12 years of age. Moreover, potential factors that might influence the association between childhood obesity and NE were used to adjust the association in a multivariable logistic regression analysis.

## Results

**Figure 1:**
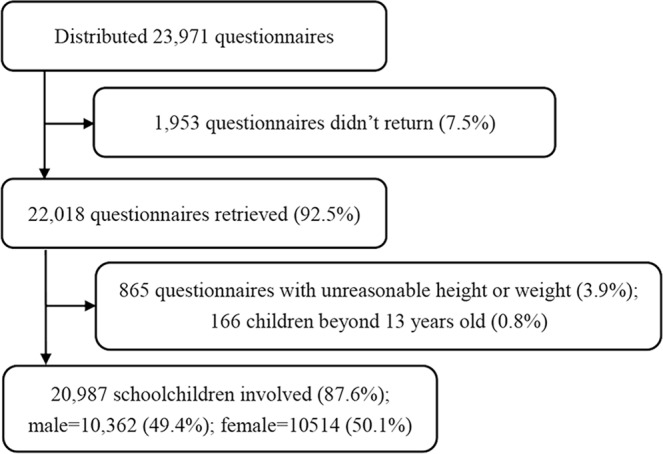
Flow chart of participation.

A total of 20,987 representative schoolchildren aged 5–12 met the selection criteria (87.6% of the screened children) and were enrolled in our survey (Fig. 1). Most of them (n = 19,639, 93.6%) were Chinese Han. Participants had a mean (SD) age of 9.17 (1.73) years; 10,362 were boys (49.4%) and 10,514 were girls (50.1%).

Sociodemographic characteristics were compared among different subgroups (Table 1). Overall, the prevalence of obesity was 1,386 (6.6%): 1,042 (16.3%) in boys and 344 (4.9%) in girls. Obese children were more likely to be younger and suffer from NE compared to children with normal body mass index (BMI). Meanwhile, boys were more likely to be obese than girls (Table 1). We divided the whole study population into six age groups: (1) 5–6 years old (n = 1,509, 11.3%); (2) 7 years old (n = 2,282, 17.0%); (3) 8 years old (n = 2,415, 18.0%); (4) 9 years old (n = 2,369, 17.7%); (5) 10 years old (n = 2,195, 16.4%); and (6) 11–12 years old (n = 2,636, 19.7%). The prevalence of obesity was 165 (10.9%), 291 (12.6%), 278 (11.5%), 269 (11.0%), 210 (9.6%), and 182 (6.9%), respectively (χ^2^ = 54.48, P < 0.001), suggesting that younger children had a higher prevalence of obesity than older ones (Table 1). Nevertheless, there were no significant differences in ADHD prevalence, parents’ education levels, house size, family structure, and household income between children with obesity and those with normal BMI (Table 1). Supplementary Table S1 presents the sociodemographic characteristics of all the subjects, including those with unreasonable BMI Z score measures.

**Table 1:** Comparison of subjects’ sociodemographic characteristics.

Characteristics	Total (N = 20,987)	Obese children (N = 1,386)	Normal (N = 12,020)	P value
Age, mean (SD)	9.21 (1.74)	8.92 (1.61)	9.24 (1.75)	<0.001
Male, n (%)	6,377 (47.6%)	1,042 (16.3%)	5,335 (83.7%)	<0.001
Female, n (%)	7,029 (52.4%)	344 (4.9%)	6,685 (95.1%)	
NE, n (%)	592 (4.4%)	79 (5.8%)	513 (4.3%)	0.013
Asthma, n (%)	421 (3.1%)	67 (4.8%)	354 (2.9%)	<0.001
ADHD, n (%)	540 (4.0%)	63 (4.6%)	477 (4.0%)	0.298
Depressive feelings, n (%)	2,149 (16.0%)	272 (19.6%)	1,877 (15.6%)	<0.001
Snoring, n (%)	1,572 (11.7%)	287 (20.7%)	1,285 (10.7)	<0.001
Maternal educational level				0.524
Illiterate	161 (1.2%)	19 (1.4%)	142 (1.2%)	
Primary or middle school	3,438 (26.1%)	367 (26.9%)	3,071 (26.0%)	
Junior high school	4,482 (34.0%)	478 (35.1%)	4,004 (33.9%)	
College or university	4,329 (32.8%)	420 (30.8%)	3,909 (33.1%)	
Master or doctor’s degree	772 (5.9%)	79 (5.8%)	693 (5.9%)	
Paternal educational level				0.644
Illiterate	88 (0.7%)	10 (0.7%)	78 (0.7%)	
Primary or middle school	2,958 (22.2%)	316 (22.9%)	2,642 (22.1%)	
Junior high school	4,659 (34.9%)	486 (35.3%)	4,173 (34.9%)	
College or university	4,269 (32.0%)	441 (32.0%)	3,828 (34.9%)	
Master or doctor’s degree	1,362 (10.2%)	125 (9.1%)	1,237 (10.3%)	
House size^a^				0.895
<15	1,911 (14.4%)	195 (14.3%)	1,716 (14.5%)	
15–25	4,180 (31.6%)	420 (30.8%)	3,760 (31.7%)	
25–35	3,397 (25.7%)	359 (26.3%)	3,038 (25.7%)	
>35	3,742 (28.3%)	389 (28.5%)	3,353 (28.3%)	
Family structure				0.155
Single-parent family	697 (5.2%)	69 (5.0%)	628 (5.2%)	
Two-parent family	8,501 (63.7%)	853 (61.6%)	7,648 (63.9%)	
Large family	4,145 (31.1%)	462 (33.4%)	3,692 (30.8%)	
Household income^b^				0.365
<125	2,507 (18.9%)	242 (17.6%)	2,265 (19.0%)	
125–235	4,426 (33.3%)	449 (32.6%)	3,977 (33.4%)	
235–390	3,190 (24.0%)	351 (25.5%)	2,839 (23.8%)	
>390	3,162 (23.8%)	335 (24.3%)	2,827 (23.7%)	
Age group				<0.001
5–6 years old	1,509 (11.3%)	165 (11.9%)	1,344 (11.2%)	
7 years old*	2,282(17.0%)	291 (21.0%)	1,991 (16.6%)	
8 years old*	2,415 (18.0%)	278 (20.1%)	2,137 (17.8%)	
9 years old	2,369(17.7%)	269 (18.8%)	2,100 (17.5%)	
10 years old	2,195 (16.4%)	210 (15.2%)	1,985 (16.5%)	
11–12 years old*	2,636 (19.7%)	182 (13.1%)	2,454 (20.4%)	

Abbreviations: NE, nocturnal enuresis; SD, standard deviation; ADHD, attention deficit/hyperactivity disorder. ^a^Measure of house size is square meters per person; ^b^measure of household income is dollars per person per month. *P < 0.05.

### Association between NE and potential influencing factors

Except obesity, snoring, asthma, ADHD, and depressive moods were associated with a higher risk of NE (Table 2). Univariable logistic regression analyses also showed that household income, house size, and family structure (or family members) were significantly associated with NE. As a result, these factors were entered in the multivariable logistic regression.

**Table 2:** The relationship between NE and risk factors by univariate logistical regression.

	β	OR	95%CIs	P values
Obesity	0.31	1.36	1.07–1.74	0.013
Snoring	1.07	2.91	2.50–3.38	<0.001
ADHD	0.95	2.58	2.06–3.22	<0.001
Depressive moods	0.56	1.75	1.50–2.03	<0.001
Asthma	0.39	1.48	1.09–2.02	0.013
Big house	−0.08	0.92	0.87–0.98	0.014
More persons at home	−0.22	0.80	0.71–0.90	<0.001
Higher income	−0.21	0.81	0.76–0.86	<0.001
Lower maternal educational level	0.05	1.05	0.97–1.13	0.186
Lower paternal educational level	0.06	1.03	0.94–1.09	0.198

Abbreviations: ADHD, attention deficit/hyperactivity disorder. Results are presented as ORs and 95%CIs.

### Association between obesity and NE

The relationship between obesity and NE was analyzed through multivariable logistic regression models (Table 3; Supplemental Table S2 for the analysis of all subjects, including those with unreasonable BMI Z score). In model 1, obesity was a risk factor for NE after adjusting for confounding factors including family structure, household income and house size [OR (95%CI) = 1.42 (1.11–1.82), P = 0.005]. After controlling for the influence of asthma in model 2, the relationship between obesity and NE did not change [OR (95%CI) = 1.4 2(1.10–1.82), P = 0.005]. In model 3, adjusting for ADHD decreased the OR slightly [OR 95%CI) = 1.41 (1.10–1.81), P = 0.006]. Obesity continued to be a significant risk factor for NE after controlling for depressive moods in model 4, even though the OR decreased [OR (95%CI) = 1.34 (1.07–1.76), P = 0.012]. In model 5, with the addition of snoring, the relationship between obesity and NE was attenuated and the OR was no longer significant [OR (95%CI) = 1.21 (0.94–1.56), P = 0.138].

**Table 3:** The relationship between NE and obesity by multivariate logistical regression.

	Model 1	Model 2	Model 3	Model 4	Model 5
Obesity	1.42 (1.11–1.82)**	1.42 (1.10–1.82)**	1.41 (1.10–1.81)**	1.38 (1.07–1.76)*	1.21 (0.94–1.56)
Asthma		1.44 (0.93–2.22)	1.28 (0.82–2.00)	1.23 (0.79–1.91)	1.11 (0.71–1.73)
ADHD			2.67 (1.99–3.58)***	2.32 (1.72–3.13)***	2.09 (1.54–2.82)***
Depressing				1.96 (1.62–2.38)***	1.89 (1.55–2.29)***
Snoring					2.89 (2.37–3.52)***

Abbreviations: ADHD, attention deficit/hyperactivity disorder. Results are presented as ORs and 95%CIs. *P < 0.05; **P < 0.01; ***P < 0.001.

**Figure 2:**
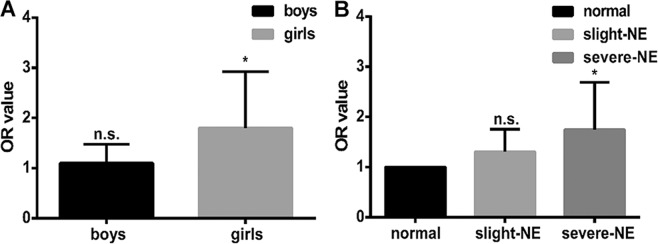
Sensitivity analysis in different genders and different NE severity. (**A**) Association of gender with NE through multivariable logistic regression after controlling for confounding factors including family structure, household income, and house size. The difference in OR between different genders was compared using an unpaired t-test, and the difference between the two subgroups was significant (P < 0.001). (**B**) Sensitivity analysis for different NE severity. Normal children were regarded as the reference group. The difference in OR between groups with different NE severity was compared by unpaired t-test, and there was statistical significance between the two subgroups (P < 0.001). n.s. not significant; *P < 0.05.

We also performed sensitivity analysis to examine the influence of gender and NE severity on the relationship between obesity and NE. It revealed that the association between obesity and NE was stronger in girls [OR (95%CI) = 1.802 (1.111–2.924), P = 0.017] compared with boys [OR (95%CI) = 1.103 (0.825–1.474), P = 0.508]. In addition, when compared to the normal group, children with severe NE [OR (95%CI) = 1.745 (1.132–2.690), P = 0.012] were at a higher risk of being obese than those with mild NE [OR (95%CI) = 1.307 (0.974–1.755), P = 0.074] (Fig. 2).

## Discussion

The aim of this study was to investigate the relationship between childhood obesity and NE in a large representative population of Chinese children. The results showed there was a positive relationship between childhood obesity and NE, but the relationship was influenced by several factors including snoring, depressive moods, and ADHD, among which snoring played the most important role.

To the best of our knowledge, this might be the first study on the potential association between childhood obesity and NE based on a nationwide sample. The results of this study strongly suggest that children with obesity are at a 1.36 times higher risk of NE compared with their peers with normal BMI, as supported by previous studies^5,10,11^. The OR decreased when adjusted for ADHD and depressive moods. In addition, snoring was used to adjust the model and the risk of NE for obese children decreased significantly. This finding was in line with another study that showed that NE and obesity were associated with obstructive sleep apnea (OSA)^8^. The prevalence of obesity was 1,386 (6.6%) in Chinese school children. There was a significant difference between boys and girls in terms of prevalence, with 1,042 (16.3%) in boys and 344 (4.9%) in girls.

The relationship between childhood obesity and NE is apparently mediated by snoring, ADHD or depressive moods. It has been reported that obstructive sleep apnea symptoms (OSAS), obesity, and childhood NE might be associated with each other^8^, and OSAS includes snoring as a clinical symptom. Numerous studies have indicated that obesity was associated with a higher occurrence of snoring^12–14^. Chronic snoring may lead to brain hypoxia^15^, and this could influence the threshold for arousal at night. Indeed, snoring children might have lower blood oxygen in the cerebral cortex which would make it more difficult to wake in response to low oxygen or a full bladder^16^. Low blood oxygen in the developing brain might also change the normal circadian rhythm of the antidiuretic hormone (ADH). It has been suggested in a number of studies that in a normal situation plasma ADH levels might increase at night to reduce urine production, and then decrease during the day^17,18^. This ADH rhythm might be absent in patients suffering from NE and previous studies have reported that a reversed ADH rhythm could in part contribute to NE in children and adolescents^19^. The negative intrathoracic pressure caused by an excessive inspiratory effort might lead to a rise in atrial natriuretic peptide in the blood, which would increase water excretion and inhibit the release of ADH^8^. Lower oxygen in the central nervous system might reduce the intensity and frequency of the “brain-bladder dialogue”, which is an important reason for NE. It has been reported that the most important cause of snoring was adenotonsillar hypertrophy, and treating NE patients with adenoidectomy for OSAS was effective^20,21^. In the present study, the OR decreased rapidly and the association was not significant when snoring was included as a factor in model 5. This result strongly suggests that snoring plays the most important role in the association between obesity and NE. Nevertheless, no formal diagnostic of OSAS was performed in the present study and we relied on snoring as a manifestation of OSAS. Further study is necessary to refine this association.

Obese children are reported to have poor mental health and psychosocial function, and they are more likely to be unsatisfied with their weight and have a depressive mood^22–25^. Obese children show worse psychological health and function, having lower self-esteem and body-esteem^26^. Children with persistent depressive moods are more likely to stay up late at night, which would affect the release of ADH at night^18,27^. Meanwhile, children with NE might feel depressed after punishment for bedwetting, and have a reported reduced quality of life^28,29^. In support of these findings, we found that in model 4, the OR decreased after including depressive moods as a factor, indicating that depressive moods might play a role in the association between NE and obesity. Nevertheless, since the present study was a cross-sectional survey, the exact cause-to-effect relationship between NE and depressive moods could not be determined.

In model 3, taking ADHD into consideration changed the OR slightly. We consider from our study that the role of ADHD in mediating the relationship between NE and obesity is not significant. Indeed, ADHD has been considered a risk factor for obesity^30–32^. Evidence has shown that children with ADHD have eating problems and bulimia nervosa (BN)^33,34^. The three cardinal syndromes including attention deficit, hyperactivity, and impulsivity are reported to be associated with obesity, as attention deficit might make the appetite over-stimulated, and impulsivity is related to many diseases such as binge eating^35^. Furthermore, studies have suggested that both ADHD and NE were associated with delayed maturation of the central nervous system^36,37^, and NE is a common co-morbidity of childhood ADHD^38,39^.

Overall, our data support the hypothesis that snoring, depressive moods, and ADHD contributed to the association between obesity and NE. Among these factors, snoring was the most important mediator.

This study has several strengths. The study population was from a nationwide survey and the result might be more representative than previous studies. Moreover, potential confounding factors including household income, house size, and family structure were adjusted in the multivariable logistic regression analysis. Nevertheless, there are several limitations that should also be considered. First, this is a cross-sectional study and the data was collected at one time point, without a follow-up. The exact mechanisms of the association between obesity and NE cannot be revealed, and further studies into the pathogenesis are needed. Second, the diagnosis of NE was based on asking about the frequency of bedwetting, so we could not exclude children with secondary causes of NE. Even if questions about possible primary causes of secondary NE were included in the questionnaire, the condition had to be diagnosed for the parents to answer that the condition was present. Third, a recall bias is possible, because the information was gathered based on the parents’ memory. More studies into the relationship between childhood obesity and NE are needed. Especially, the association between the distribution of body fat and NE could be examined.

In conclusion, the present study strongly suggests that childhood obesity is a risk factor for NE. This association is attributed to snoring, depressive moods, and ADHD. Among these factors, snoring might play the most important role.

## Subjects and Methods

### Subjects

In November and December 2005, a nationwide, cross-sectional study of the sleep arrangements, patterns, and behaviors of schoolchildren was conducted^40^. Eight Chinese cities (Urumqi, Chengdu, Xi’an, Hohhot, Wuhan, Canton, Shanghai, and Harbin) were selected using cluster stratification based on geographic location, density of population, and economy, as described previously^40^. Within every city, 3–10 districts were selected randomly and 1–2 primary schools were selected in every district. Finally, there were 55 elementary schools from both urban and rural areas. In total, 23,971 schoolchildren in these schools were enrolled. We distributed the questionnaire to the children and their parents, and 22,018 (92.5%) completed questionnaires were returned. Questionnaires with more than 67% questions uncompleted were considered as invalid^41^.

The exclusion criteria were: (1) children (n = 865, 3.9%) with unreasonable measurement that could extremely affect the analysis and result: BMI Z score >3 or <−3 [Z = (X-mean)/standard deviation]; (2) children (n = 166, 0.8%) above 13 years old were excluded in order to eliminate the influence of adolescence; or (3) children (n = 0) with additional chronic renal diseases and other diseases that would cause secondary nocturnal enuresis (the questionnaire included questions about chronic renal diseases, diabetes, central diabetes insipidus, chronic lower urinary tract infections, and others).

After obtaining ethical approval from the Human Research Ethics Committee of Shanghai Jiao Tong University School of Medicine, we informed participants and their caregivers about our survey, and they signed informed consent. All methods were performed in accordance with the relevant guidelines and regulations. Meanwhile, they were informed that the whole survey involvement would be confidential and voluntary, and they were also reassured about their right to withdraw from the survey at any time.

Height and weight of the children were measured by teachers trained in healthcare in each school. We used the World Health Organization (WHO) growth reference charts to determine the BMI and nutritional status for each child^42^. The cut-offs for overweight and obesity were determined as the age- and sex-specific 85^th^ and 95^th^ percentiles of BMI, respectively. As validation, the cut-offs of the Chinese Center for Disease Control and Prevention (CDC) growth charts showed consistent results. It was worth mentioning that children who were underweight or overweight were not included in the final analysis.

According to the Diagnostic and Statistical Manual of Mental Disorders, Fifth Edition, (DSM-V), NE was defined as repeated bedwetting twice a week in at least three consecutive months in the absence of any related physical disorder or medical condition^43^. In our study, a child wetting his bed at least twice a week was diagnosed with NE, while this was considered severe if the frequency was more than five times a week. According to this, participants in our study were divided into two groups: with and without NE, and then those with NE were subgrouped as mild NE and severe NE.

### Questionnaires

The questionnaire contained three sections. The first section was designed by the author and included information about the family and social environment, and the demographic characteristics of the subject. The second section was the Chinese version of the Children’s Sleep Habits Questionnaire that describes the sleeping arrangements, patterns, and behaviors of the child. This questionnaire has been shown to have good validity and reliability when evaluating sleep in both American and Chinese children^44,45^. The third section represented the quality of life of every child. In this section, we collected information about the children’s quality of life, moods and emotions.

The sociodemographic characteristics section consisted of the children’s age, gender, grade, ethnic background, height, weight, parents’ educational levels (illiterate, primary or middle school, junior high school, college and university, or above university degree), household income (<125, 125–235, 235–390, or >390, dollars per person per month), house size (<15, 15–25, 25–35, or >35 square meters per person), and the family structure (classified by single-parent family, two-parent family, and large family with grandparents). Based on the child’s height and weight, the BMI was calculated as: weight (kg)/height (m)^2^, and this was used to calculate the BMI Z scores.

Teachers and parents involved in our study were trained and informed about the standard method to measure the height and weight. Each subject’s height was measured by their teachers, without shoes, and weight was measured while wearing light clothes without shoes and hair accessories at school. To assess the obesity and related risk factors, the following questions were presented to the parents: (1) does the child snores while sleeping at night, and answers were described with three points: usually, referring to 5–7 days a week; sometimes, referring to 2–4 days a week; and occasionally, referring to 0–1 days a week; (2) does the child feel depressed while studying: yes or no; (3) was a professional diagnosis of ADHD made for the child: yes or no; and (4) was a professional diagnosis of asthma made for the child: yes or no.

The situation regarding bedwetting was evaluated through asking the parents: (1) does the child wet his/her bed during sleep, answers were according to a 3-point Likert scale: usually, referring to 5–7 days a week; sometimes, referring to 2–4 days a week; and occasionally, referring to 0–1 days a week, but they were recorded into two items: yes for usually and sometimes, no for occasionally; (2) did the parent, sibling, or grandparent have bedwetting, the answer was yes or no.

### Statistical analysis

First, we used the Kolmogorov-Smirnov test to assess the data for normal distribution. Then the sociodemographic characteristics were described using total number and prevalence for categorical data or mean and standard deviation for continuous data. The unpaired t-test was applied to compare the difference of age and BMI between schoolchildren with or without obesity. The chi-square test was conducted to compare the gender, parents’ educational level, household income, family structure, prevalence of NE, prevalence of snoring, asthma, and depressive moods between the different groups. In order to decide on the variables that might influence the relationship between obesity and NE, we first conducted univariable logistic regression, and then multivariable logistic regression analysis to identify the association between obesity with NE. The process was conducted in the following order: first, obesity and confounding factors identified in the univariable logistic regression analyses were entered into model 1. Second, besides obesity and the confounding factors, asthma was added in model 2. In addition to the variables in model 2, we also took ADHD into consideration in model 3. In model 4, we adjusted for the influence of depressive moods and added all variables in model 3. In model 5, snoring was added on the basis of model 4 (age and gender were not included as they were taken into consideration when determining the diagnosis of obesity). The results were presented as odds ratio (OR) and 95% confidence intervals (CIs). Additional analyses have been conducted in a study sample in which all children were included, even those with unreasonable measures (BMI Z score <−3 or >3). All analyses were performed with the Statistical Program for Social Science software (SPSS, version 19.0, IBM Corp., USA), and P < 0.05 was considered to be statistically significant (two tailed).

## Supplementary information

Supplementary Dataset 1
