# Supplementary material for: Nocturnal enuresis in obese children: a nation-wide epidemiological study from China
Source: Sci Rep. 2019 Jun 10;9:8414. doi: 10.1038/s41598-019-44532-5 (PMC6557885; doi:10.1038/s41598-019-44532-5)
Supplement: Supplementary file 1 — Supplementary Dataset 1 [file 41598_2019_44532_MOESM1_ESM.docx]

**Nocturnal enuresis in obese children: a nation-wide epidemiological study from China**

**Running title:** Obesity and nocturnal enuresis

AnyiZhang^1,2,3^, Shenghui Li^4^, Yiwen Zhang^1,2,3^, Fan Jiang^1,2,3^, Xingming Jin^1,2,3^, Jun Ma^1,2,3,*^

^1^Department of Developmental and Behavioral Pediatrics, Shanghai Children’s Medical Center, Shanghai Jiao Tong University School of Medicine, Shanghai, China

^2^Shanghai Institute of Pediatric Translational Medicine, Shanghai Children’s Medical Center, Shanghai Jiao Tong University School of Medicine, Shanghai, China

^3^MOE-Shanghai Key Laboratory of Children’s Environmental Health, Shanghai Jiao Tong University School of Medicine, Shanghai, China

^4^Shanghai Jiao Tong University School of Medicine, Shanghai, China

**^*^Corresponding Author:**

Jun Ma

Department of Developmental and Behavioral Pediatrics, Shanghai Children’s Medical Center, Shanghai Jiao Tong University School of Medicine, Shanghai, China

Shanghai Institute of Pediatric Translational Medicine, Shanghai Children’s Medical Center, Shanghai Jiao Tong University School of Medicine, Shanghai, China

MOE-Shanghai Key Laboratory of Children’s Environmental Health, Shanghai Jiao Tong University School of Medicine, Shanghai, China

Tel: +86-13917230745

Fax: +86-21-57643271

E-mail: [majun@shsmu.edu.cn](mailto:majun@shsmu.edu.cn)

**Supplement Table 1**. Comparison of new subjects’ (with unreasonable BMIZ score measures) sociodemographic characteristics.

| Characteristics | Total  (N=21,852) | Obese patients  (N=2,235) | Normal  (N=12,025) | P value |
| --- | --- | --- | --- | --- |
| Age mean (SD) | 9.17(1.73) | 8.83(1.61) | 9.24(1.75) | <0.001 |
| Male n (%) | 6,795(47.7%) | 1,459(21.5%) | 5,336(78.5%) | <0.001 |
| Female n (%) | 7,465(52.3%) | 776(10.4%) | 6,689(89.6%) |  |
| NE n (%) | 647(4.6%) | 134(6.0%) | 513(4.3%) | <0.001 |
| Asthma n (%) | 447(3.1%) | 93(4.2%) | 354(2.9%) | 0.002 |
| ADHD n (%) | 581(4.1%) | 104 (4.7%) | 477(4.0%) | 0.130 |
| Depressive feelings n (%) | 2,303(16.2%) | 426 (19.1%) | 1,877(15.6%) | <0.001 |
| Snoring n (%) | 1,693(11.7%) | 408(18.3%) | 1,285(10.7) | <0.001 |
| Maternal educational level |  |  |  | <0.001 |
| Illiterate | 179(1.3%) | 37(1.7%) | 142(1.2%) |  |
| Primary or middle school* | 3,715(26.5%) | 642(29.2%) | 3,073(26.0%) |  |
| Junior high school | 4,763(34.0%) | 758(34.5%) | 4,005(33.9%) |  |
| College or university* | 4,554(32.5%) | 643(29.3%) | 3,911(33.1%) |  |
| Master or doctor’s degree | 809(5.8%) | 116(5.3%) | 693(5.9%) |  |
| Paternal educational level |  |  |  | 0.023 |
| Illiterate | 99(0.7%) | 21(0.9%) | 78(0.7%) |  |
| Primary or middle school* | 3,179(22.4%) | 534(24.1%) | 2,645(22.1%) |  |
| Junior high school | 4,965(35.0%) | 791(35.7%) | 4,174(34.9%) |  |
| College or university | 4,501(31.7%) | 672(30.3%) | 3,829(32.0%) |  |
| Master or doctor’s degree* | 1,435(10.1%) | 198(8.9%) | 1,237(10.3%) |  |
| House size ^a^ |  |  |  | 0.269 |
| <15 | 2,054(14.6%) | 336(15.3%) | 1,718(14.5%) |  |
| 15-25 | 4,416(31.4%) | 654(29.8%) | 3,762(31.7%) |  |
| 25-35 | 3,598(25.6%) | 559(25.5%) | 3,039(25.6%) |  |
| >35 | 3,999(28.4%) | 646(29.4%) | 3,353(28.2%) |  |
| Family structure |  |  |  | 0.056 |
| Single-parent family | 758(5.3%) | 129(5.8%) | 629(5.3%) |  |
| Two-parent family | 9,017(63.5%) | 1,365(61.3%) | 7,652(63.9%) |  |
| Large family | 4,426(31.2%) | 734(32.9%) | 3,692(30.8%) |  |
| Household income ^b^ |  |  |  | 0.841 |
| <125 | 2,707(19.2%) | 439(19.8%) | 2,268(19.0%) |  |
| 125-235 | 4,713(33.4%) | 736(33.3%) | 3,977(33.4%) |  |
| 235-390 | 3,364(23.8%) | 523(23.6%) | 2,841(23.8%) |  |
| >390 | 3,342(23.7%) | 515(23.3%) | 2,827(23.7%) |  |
| Age group |  |  |  | <0.001 |
| 5-6 years old* | 1,603 (11.4%) | 286 (12.8%) | 1,344 (11.2%) |  |
| 7 years old* | 2,502 (17.5%) | 510 (22.8%) | 1,992 (16.6%) |  |
| 8 years old* | 2,585 (18.1%) | 448 (20.0%) | 2,137 (17.8%) |  |
| 9 years old | 2,499 (17.5%) | 388 (17.4%) | 2,111 (17.8%) |  |
| 10 years old | 2,330 (16.3%) | 345 (15.4%) | 1,985 (16.5%) |  |
| 11-12 years old* | 2,714 (19.0%) | 258 (11.5%) | 2,456 (20.4%) |  |

Abbreviations: NE, nocturnal enuresis; SD, standard deviation; ADHD, attention deficit/hyperactivity disorder

a, measure of house size is square meters per person; b, measure of household income is dollars per person per month.* P＜0.05.

**Table 2**. The relationship between NE and obesity by multivariate logistical regression.

|  | Model 1 | Model 2 | Model 3 | Model 4 | Model 5 |
| --- | --- | --- | --- | --- | --- |
| Obesity | 1.48(1.21-1.81)*** | 1.47(1.21-1.80)*** | 1.47(1.20-1.79)*** | 1.44(1.18-1.75)*** | 1.21(0.97-1.50) |
| Asthma |  | 1.49(0.99-2.22) | 1.35(0.89-2.01) | 1.28(0.84-1.95) | 1.16(0.76-1.77) |
| ADHD |  |  | 2.59(1.96-3.44)*** | 2.25(1.69-2.99)*** | 2.04(1.53-2.72)*** |
| Depressing |  |  |  | 2.04(1.70-2.45)*** | 1.98(1.65-2.38)*** |
| Snoring |  |  |  |  | 2.83(2.35-3.42)*** |

Abbreviations: ADHD, attention deficit/hyperactivity disorder. Results are presented as ORs and 95%CIs. We adjusted the potential confounding factors including house size, household income and family structure. *** P＜0.001
